# The Picture Magazine

**Published:** 1893-01-28

**Authors:** 


### "THE PICTURE MAGAZINE"

FROM the busy office of the "Strand Magazine," "The Picture Magazine" has been launched forth into the world of periodicals. Doubtless, Mr. Newnes keeps a careful finger on the public pulse, and feels assured of the popularity of such a venture, or he would hardly have undertaken so costly a form of production. He may foresee in the future that the army of readers of light literature are to be divided into two very distinct sections—those who indulge in the minutest and most analytical of mind studies, and others who shrink from troubling themselves with problems which sight will not convey by easy roads to their understanding. To the latter "The Picture Magazine" will be very welcome. It aims at instructing as well as amusing. The range, indeed, of the illustrations is very wide, and they are grouped under Fine Arts, Comic Pictures, Portraits, Pictures for Children, Pictures of Places, and Miscellaneous Pictures. The pictures are carefully reproduced, and the whole magazine, which is a monthly one, consists of 60 pages. The price being 6d., we can quite imagine that "The Picture Magazine" will be popular in the hospital and sick-room.

For Student of Medicine and Medical Appointments see pages VIII. and IX. overleaf.

